# 2-[4-(4-Methoxy­phen­yl)-5-(2-pyrid­yl)-4*H*-1,2,4-triazol-3-yl]phenol

**DOI:** 10.1107/S1600536809022223

**Published:** 2009-06-27

**Authors:** Mei-An Zhu, Zhao-Di Liu, Shu-Ping Zhang, Ying Wei, Si-Chang Shao

**Affiliations:** a Department of Chemistry, Fuyang Normal College, Fuyang Anhui 236041, People’s Republic of China

## Abstract

In the title compound, C_20_H_16_N_4_O_2_, the benzene rings of the 2-hydroxy­phenyl and 4-methoxy­lphenyl groups form dihedral angles of 64.02 (8) and 77.39 (7)°, respectively, with the mean plane of the triazole ring. The dihedral angle between the triazole ring mean plane and the pyridyl ring is 9.61 (8)°. In the crystal, inter­molecular N—H⋯O hydrogen bonds link the mol­ecules into zigzag chains propagating in [010].

## Related literature

For the potential anti­fungal and anti­bacterial properties of 1,2,4-triazoles, see: Collin, *et al.* (2003 ▶); Papakonstanti­nou-Garoufalias, *et al.* (2002 ▶). For the synthesis of the title compound, see: Zhang *et al.* (2004 ▶). For related structures, see: Zhang *et al.* (2004 ▶); Zhang, Liu, Ma *et al.* (2005 ▶); Zhang, Liu, Yang *et al.* (2005 ▶); Zhu *et al.* (2000 ▶).
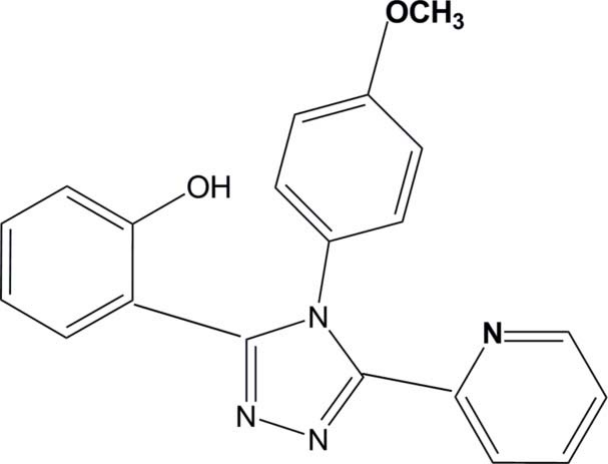


## Experimental

### 

#### Crystal data

C_20_H_16_N_4_O_2_
*M* *_r_* = 344.37
Monoclinic, 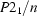

*a* = 10.0842 (9) Å
*b* = 10.4903 (9) Å
*c* = 16.7214 (14) Å
β = 94.658 (2)°
*V* = 1763.1 (3) Å^3^
*Z* = 4
Mo *K*α radiation
μ = 0.09 mm^−1^
*T* = 293 K
0.10 × 0.10 × 0.08 mm

#### Data collection

Bruker SMART CCD area-detector diffractometer
Absorption correction: multi-scan (*SADABS*; Sheldrick, 1996 ▶) *T*_min_ = 0.991, *T*_max_ = 0.993
8997 measured reflections
3278 independent reflections
2734 reflections with *I* > 2σ(*I*)
*R*_int_ = 0.018

#### Refinement

*R*[*F* ^2^ > 2σ(*F* ^2^)] = 0.038
*wR*(*F* ^2^) = 0.110
*S* = 1.04
3278 reflections
238 parameters
H atoms treated by a mixture of independent and constrained refinement
Δρ_max_ = 0.27 e Å^−3^
Δρ_min_ = −0.13 e Å^−3^

### 

Data collection: *SMART* (Siemens, 1996 ▶); cell refinement: *SAINT* (Siemens, 1996 ▶); data reduction: *SAINT*; program(s) used to solve structure: *SHELXS97* (Sheldrick, 2008 ▶); program(s) used to refine structure: *SHELXL97* (Sheldrick, 2008 ▶); molecular graphics: *SHELXTL* (Sheldrick, 2008 ▶); software used to prepare material for publication: *SHELXTL*.

## Supplementary Material

Crystal structure: contains datablocks global, I. DOI: 10.1107/S1600536809022223/su2120sup1.cif

Structure factors: contains datablocks I. DOI: 10.1107/S1600536809022223/su2120Isup2.hkl

Additional supplementary materials:  crystallographic information; 3D view; checkCIF report

## Figures and Tables

**Table 1:** Hydrogen-bond geometry (Å, °)

*D*—H⋯*A*	*D*—H	H⋯*A*	*D*⋯*A*	*D*—H⋯*A*
O1—H1*O*⋯N1^i^	0.938 (18)	1.759 (19)	2.6937 (16)	174.2 (16)

Symmetry code: (i) 
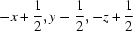
.

**Fig. 1.**
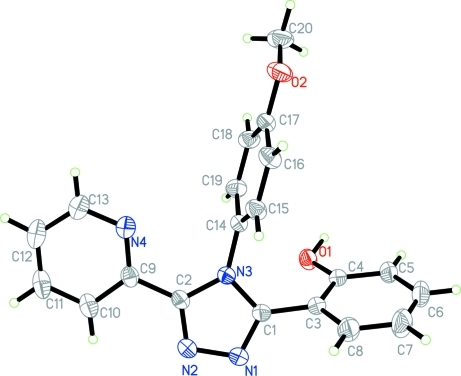
A view of the molecular structure of the title compound, showing 30% probability displacement ellipsoids and the atom-numbering scheme.

**Fig. 2.**
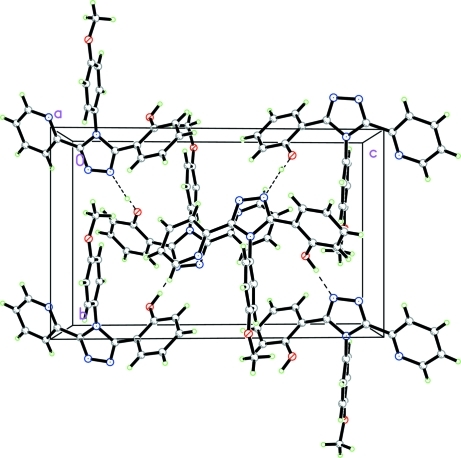
A view along the a axis of the crystal apckinh i nthe title compound. The intermolecular N-H···O hydrogen bonds are shown dashed lines (details are given in Table 1).

## supplementary crystallographic information

### Comment

Molecules containing a 1,2,4-triazole moiety have elicited considerable interest among medicinal chemists because they display a wide range of antifungal (Collin *et al.*,  2003) and antibacterial (Papakonstantinou-Garoufalias *et al.*, 2002) activities. We have synthesed and reported the crystal structures of various 1,2,4-triazole ligands and their metal complexes (Zhang *et al.*, 2004; Zhang, Liu, Ma *et al.*, 2005; Zhang, Liu, Yang *et al.*, 2005; Zhu *et al.*, 2000). As an extension of our work on the structural characterization of triazole derivatives, we report herein on the crystal structure of the title compound.

In the title compound the pyridyl ring and the benzene rings lie in a propeller arrangement around the central 1,2,4-triazole ring (Fig. 1), thereby minimizing the steric effects among these rings. The benzene rings of the 2-hydroxyphenyl and 4-methoxylphenyl groups are inclined to the mean plane of the triazole ring by 64.02 (8) and 77.39 (7)°, respectively. In contrast the pyridyl ring is inclined to the triazole ring by 9.61 (8)°.

In the crystal structure intermolecular O–H···N hydrogen bonds, involving hydroxyl O1-H1O and a triazole N-atom, N1, link the molecules into zigzag chains propagating in the [010] direction (Fig. 2 and Table 1).

### Experimental

The title compound was synthesized according to a literature method (Zhang *et al.*, 2004). Equivalent amounts of *p*-methoxylphenylphosphazoanilide and *N*-(2-pyridoyl)-*N*'-(2-Hydroxyphenyl)hydrazine were reacted in *N*,*N*'-dimethylaniline for 3 h at 463 K, with stirring. Colourless block-shaped crystals were obtained by slow evaporation of an acetone solution. The crystals were collected and dried in a vacuum desiccator using anhydrous CaCl_2_ (yield 52%).

### Refinement

The hydroxyl H-atom was located in a difference Fourier map and freely refined, O-H = 0.938 (18)Å, with *U*_iso_(H) = 1.2 *U*_eq_(O). The C-bound H atoms were placed in geometrically idealized positions and treated as riding atoms: C—H = 0.93–0.96 Å, with *U*_iso_(H) = 1.2 or 1.5 (methyl) *U*_eq_(C).

### Crystal data

**Table:**

C_20_H_16_N_4_O_2_	*F*(000) = 720
*M**_r_* = 344.37	*D*_x_ = 1.297 Mg m^−^^3^
Monoclinic, *P*2_1_/*n*	Mo *K*α radiation, λ = 0.71073 Å
Hall symbol: -P 2yn	Cell parameters from 2.3 reflections
*a* = 10.0842 (9) Å	θ = 15–532°
*b* = 10.4903 (9) Å	µ = 0.09 mm^−^^1^
*c* = 16.7214 (14) Å	*T* = 293 K
β = 94.658 (2)°	Block, colourless
*V* = 1763.1 (3) Å^3^	0.10 × 0.10 × 0.08 mm
*Z* = 4	

### Data collection

**Table:**

Bruker SMART CCD area-detector diffractometer	3278 independent reflections
Radiation source: fine-focus sealed tube	2734 reflections with *I* > 2σ(*I*)
graphite	*R*_int_ = 0.018
φ and ω scans	θ_max_ = 25.5°, θ_min_ = 2.3°
Absorption correction: multi-scan (*SADABS*; Sheldrick, 1996)	*h* = −12→9
*T*_min_ = 0.991, *T*_max_ = 0.993	*k* = −12→12
8997 measured reflections	*l* = −20→19

### Refinement

**Table:**

Refinement on *F*^2^	Primary atom site location: structure-invariant direct methods
Least-squares matrix: full	Secondary atom site location: difference Fourier map
*R*[*F*^2^ > 2σ(*F*^2^)] = 0.038	Hydrogen site location: inferred from neighbouring sites
*wR*(*F*^2^) = 0.110	H atoms treated by a mixture of independent and constrained refinement
*S* = 1.04	*w* = 1/[σ^2^(*F*_o_^2^) + (0.0578*P*)^2^ + 0.2618*P*] where *P* = (*F*_o_^2^ + 2*F*_c_^2^)/3
3278 reflections	(Δ/σ)_max_ = 0.001
238 parameters	Δρ_max_ = 0.27 e Å^−^^3^
0 restraints	Δρ_min_ = −0.13 e Å^−^^3^

### Special details

**Table:**

Geometry. All e.s.d.'s (except the e.s.d. in the dihedral angle between two l.s. planes) are estimated using the full covariance matrix. The cell e.s.d.'s are taken into account individually in the estimation of e.s.d.'s in distances, angles and torsion angles; correlations between e.s.d.'s in cell parameters are only used when they are defined by crystal symmetry. An approximate (isotropic) treatment of cell e.s.d.'s is used for estimating e.s.d.'s involving l.s. planes.
Refinement. Refinement of *F*^2^ against ALL reflections. The weighted *R*-factor *wR* and goodness of fit *S* are based on *F*^2^, conventional *R*-factors *R* are based on *F*, with *F* set to zero for negative *F*^2^. The threshold expression of *F*^2^ > σ(*F*^2^) is used only for calculating *R*-factors(gt) *etc*. and is not relevant to the choice of reflections for refinement. *R*-factors based on *F*^2^ are statistically about twice as large as those based on *F*, and *R*- factors based on ALL data will be even larger.

### Fractional atomic coordinates and isotropic or equivalent isotropic displacement parameters (Å^2^)

**Table:**

	*x*	*y*	*z*	*U*_iso_*/*U*_eq_	
O1	0.25887 (10)	0.88801 (10)	0.27051 (6)	0.0540 (3)	
H1O	0.2548 (16)	0.8171 (17)	0.3044 (10)	0.065*	
O2	0.57961 (12)	0.52684 (10)	0.08768 (7)	0.0657 (3)	
N3	0.30394 (11)	0.98466 (10)	0.09726 (6)	0.0413 (3)	
N1	0.26339 (12)	1.17680 (11)	0.13995 (7)	0.0495 (3)	
N2	0.20046 (12)	1.16372 (11)	0.06409 (7)	0.0502 (3)	
C10	0.07912 (15)	1.07145 (17)	−0.08501 (9)	0.0592 (4)	
H10	0.0505	1.1495	−0.0664	0.071*	
C1	0.32455 (13)	1.06948 (12)	0.15894 (8)	0.0430 (3)	
C2	0.22553 (13)	1.04857 (13)	0.03920 (8)	0.0430 (3)	
C3	0.40733 (14)	1.04578 (12)	0.23412 (8)	0.0459 (3)	
C4	0.37258 (14)	0.95349 (12)	0.28844 (8)	0.0448 (3)	
C5	0.45291 (16)	0.93499 (15)	0.35907 (10)	0.0595 (4)	
H5	0.4300	0.8741	0.3959	0.071*	
C6	0.5664 (2)	1.00676 (19)	0.37443 (12)	0.0780 (6)	
H6	0.6202	0.9934	0.4215	0.094*	
C7	0.6009 (2)	1.0981 (2)	0.32077 (13)	0.0868 (6)	
H7	0.6778	1.1462	0.3315	0.104*	
C8	0.52135 (18)	1.11758 (16)	0.25158 (11)	0.0686 (5)	
H8	0.5442	1.1800	0.2157	0.082*	
C9	0.17333 (13)	1.00019 (14)	−0.03971 (8)	0.0450 (3)	
C11	0.02873 (17)	1.0247 (2)	−0.15797 (10)	0.0722 (5)	
H11	−0.0347	1.0707	−0.1895	0.087*	
C12	0.07285 (19)	0.9101 (2)	−0.18364 (10)	0.0733 (5)	
H12	0.0396	0.8760	−0.2326	0.088*	
C13	0.1670 (2)	0.84634 (19)	−0.13575 (11)	0.0738 (5)	
H13	0.1972	0.7686	−0.1539	0.089*	
N4	0.21866 (15)	0.88858 (13)	−0.06456 (8)	0.0636 (4)	
C14	0.37019 (13)	0.86290 (12)	0.09457 (8)	0.0405 (3)	
C15	0.50296 (14)	0.86186 (13)	0.08103 (9)	0.0490 (3)	
H15	0.5472	0.9380	0.0730	0.059*	
C16	0.57011 (15)	0.74766 (14)	0.07936 (9)	0.0537 (4)	
H16	0.6597	0.7466	0.0697	0.064*	
C17	0.50458 (15)	0.63460 (13)	0.09194 (8)	0.0473 (3)	
C18	0.37133 (16)	0.63626 (13)	0.10599 (9)	0.0517 (4)	
H18	0.3269	0.5604	0.1145	0.062*	
C19	0.30437 (14)	0.75134 (13)	0.10732 (9)	0.0487 (3)	
H19	0.2148	0.7530	0.1169	0.058*	
C20	0.5210 (2)	0.40928 (15)	0.10653 (11)	0.0757 (5)	
H20A	0.5837	0.3416	0.1011	0.114*	
H20B	0.4433	0.3947	0.0706	0.114*	
H20C	0.4962	0.4117	0.1607	0.114*	

### Atomic displacement parameters (Å^2^)

**Table:**

	*U*^11^	*U*^22^	*U*^33^	*U*^12^	*U*^13^	*U*^23^
O1	0.0524 (6)	0.0530 (6)	0.0546 (6)	−0.0087 (5)	−0.0074 (5)	0.0118 (5)
O2	0.0727 (7)	0.0476 (6)	0.0768 (8)	0.0185 (5)	0.0054 (6)	0.0077 (5)
N3	0.0447 (6)	0.0357 (6)	0.0428 (6)	0.0005 (5)	−0.0015 (5)	0.0027 (5)
N1	0.0594 (7)	0.0384 (6)	0.0495 (7)	0.0038 (5)	−0.0027 (5)	0.0024 (5)
N2	0.0566 (7)	0.0435 (7)	0.0494 (7)	0.0054 (5)	−0.0018 (5)	0.0059 (5)
C10	0.0550 (9)	0.0737 (11)	0.0482 (9)	0.0099 (8)	−0.0007 (7)	0.0109 (7)
C1	0.0468 (7)	0.0357 (7)	0.0459 (7)	−0.0020 (6)	0.0001 (6)	0.0013 (6)
C2	0.0428 (7)	0.0424 (7)	0.0434 (7)	0.0009 (6)	0.0010 (6)	0.0079 (6)
C3	0.0521 (8)	0.0375 (7)	0.0467 (8)	−0.0003 (6)	−0.0047 (6)	−0.0016 (6)
C4	0.0483 (7)	0.0374 (7)	0.0475 (8)	0.0017 (6)	−0.0040 (6)	−0.0023 (6)
C5	0.0697 (10)	0.0540 (9)	0.0518 (9)	−0.0031 (8)	−0.0131 (7)	0.0063 (7)
C6	0.0815 (12)	0.0758 (12)	0.0699 (12)	−0.0116 (10)	−0.0351 (10)	0.0067 (9)
C7	0.0815 (13)	0.0816 (13)	0.0905 (14)	−0.0333 (11)	−0.0346 (11)	0.0112 (11)
C8	0.0723 (11)	0.0572 (10)	0.0732 (11)	−0.0205 (8)	−0.0137 (9)	0.0104 (8)
C9	0.0423 (7)	0.0515 (8)	0.0412 (7)	−0.0030 (6)	0.0028 (6)	0.0075 (6)
C11	0.0587 (10)	0.1077 (15)	0.0486 (9)	0.0108 (10)	−0.0058 (7)	0.0141 (9)
C12	0.0701 (11)	0.1032 (15)	0.0449 (9)	−0.0093 (11)	−0.0068 (8)	−0.0029 (9)
C13	0.0857 (12)	0.0764 (12)	0.0565 (10)	0.0041 (10)	−0.0109 (9)	−0.0122 (9)
N4	0.0733 (9)	0.0618 (8)	0.0530 (8)	0.0076 (7)	−0.0120 (6)	−0.0052 (6)
C14	0.0457 (7)	0.0363 (7)	0.0383 (7)	0.0027 (5)	−0.0030 (5)	0.0008 (5)
C15	0.0461 (8)	0.0426 (7)	0.0572 (9)	−0.0031 (6)	−0.0017 (6)	0.0079 (6)
C16	0.0435 (8)	0.0528 (9)	0.0644 (10)	0.0047 (6)	0.0018 (7)	0.0079 (7)
C17	0.0565 (8)	0.0433 (8)	0.0413 (7)	0.0106 (6)	−0.0003 (6)	0.0035 (6)
C18	0.0645 (9)	0.0361 (7)	0.0550 (9)	−0.0045 (6)	0.0089 (7)	0.0023 (6)
C19	0.0475 (8)	0.0430 (8)	0.0561 (9)	−0.0011 (6)	0.0077 (6)	−0.0005 (6)
C20	0.1210 (16)	0.0431 (9)	0.0652 (11)	0.0185 (9)	0.0206 (10)	0.0094 (8)

### Geometric parameters (Å, °)

**Table:**

O1—C4	1.3494 (17)	C7—H7	0.9300
O1—H1O	0.938 (18)	C8—H8	0.9300
O2—C17	1.3654 (16)	C9—N4	1.3354 (19)
O2—C20	1.414 (2)	C11—C12	1.364 (3)
N3—C1	1.3653 (17)	C11—H11	0.9300
N3—C2	1.3757 (16)	C12—C13	1.366 (3)
N3—C14	1.4439 (16)	C12—H12	0.9300
N1—C1	1.3103 (17)	C13—N4	1.335 (2)
N1—N2	1.3783 (16)	C13—H13	0.9300
N2—C2	1.3089 (18)	C14—C19	1.3706 (18)
C10—C11	1.373 (2)	C14—C15	1.3759 (19)
C10—C9	1.385 (2)	C15—C16	1.378 (2)
C10—H10	0.9300	C15—H15	0.9300
C1—C3	1.4725 (19)	C16—C17	1.382 (2)
C2—C9	1.4708 (19)	C16—H16	0.9300
C3—C8	1.386 (2)	C17—C18	1.383 (2)
C3—C4	1.3918 (19)	C18—C19	1.3843 (19)
C4—C5	1.390 (2)	C18—H18	0.9300
C5—C6	1.376 (2)	C19—H19	0.9300
C5—H5	0.9300	C20—H20A	0.9600
C6—C7	1.377 (3)	C20—H20B	0.9600
C6—H6	0.9300	C20—H20C	0.9600
C7—C8	1.369 (2)		
			
C4—O1—H1O	110.4 (10)	C10—C9—C2	118.95 (14)
C17—O2—C20	117.84 (13)	C12—C11—C10	119.19 (16)
C1—N3—C2	105.01 (11)	C12—C11—H11	120.4
C1—N3—C14	123.90 (11)	C10—C11—H11	120.4
C2—N3—C14	130.54 (11)	C11—C12—C13	118.43 (17)
C1—N1—N2	108.06 (11)	C11—C12—H12	120.8
C2—N2—N1	107.35 (11)	C13—C12—H12	120.8
C11—C10—C9	118.76 (17)	N4—C13—C12	124.21 (18)
C11—C10—H10	120.6	N4—C13—H13	117.9
C9—C10—H10	120.6	C12—C13—H13	117.9
N1—C1—N3	109.70 (12)	C9—N4—C13	116.73 (14)
N1—C1—C3	125.10 (12)	C19—C14—C15	120.59 (12)
N3—C1—C3	125.16 (12)	C19—C14—N3	121.29 (12)
N2—C2—N3	109.89 (12)	C15—C14—N3	118.10 (11)
N2—C2—C9	122.65 (12)	C14—C15—C16	119.78 (13)
N3—C2—C9	127.46 (12)	C14—C15—H15	120.1
C8—C3—C4	119.28 (13)	C16—C15—H15	120.1
C8—C3—C1	119.43 (13)	C15—C16—C17	120.08 (13)
C4—C3—C1	121.29 (12)	C15—C16—H16	120.0
O1—C4—C5	122.93 (13)	C17—C16—H16	120.0
O1—C4—C3	117.61 (12)	O2—C17—C16	115.38 (13)
C5—C4—C3	119.43 (13)	O2—C17—C18	124.72 (13)
C6—C5—C4	120.02 (15)	C16—C17—C18	119.89 (12)
C6—C5—H5	120.0	C17—C18—C19	119.72 (13)
C4—C5—H5	120.0	C17—C18—H18	120.1
C5—C6—C7	120.64 (16)	C19—C18—H18	120.1
C5—C6—H6	119.7	C14—C19—C18	119.94 (13)
C7—C6—H6	119.7	C14—C19—H19	120.0
C8—C7—C6	119.54 (16)	C18—C19—H19	120.0
C8—C7—H7	120.2	O2—C20—H20A	109.5
C6—C7—H7	120.2	O2—C20—H20B	109.5
C7—C8—C3	121.08 (16)	H20A—C20—H20B	109.5
C7—C8—H8	119.5	O2—C20—H20C	109.5
C3—C8—H8	119.5	H20A—C20—H20C	109.5
N4—C9—C10	122.68 (14)	H20B—C20—H20C	109.5
N4—C9—C2	118.37 (12)		

### Hydrogen-bond geometry (Å, °)

**Table:**

*D*—H···*A*	*D*—H	H···*A*	*D*···*A*	*D*—H···*A*
O1—H1O···N1^i^	0.938 (18)	1.759 (19)	2.6937 (16)	174.2 (16)

Symmetry codes: (i) −*x*+1/2, *y*−1/2, −*z*+1/2.
